# Intelligent Context-Aware and Adaptive Interface for Mobile LBS

**DOI:** 10.1155/2015/489793

**Published:** 2015-09-20

**Authors:** Jiangfan Feng, Yanhong Liu

**Affiliations:** ^1^College of Computer Science and Technology, Chongqing University of Posts and Telecommunications, Chongqing 400065, China; ^2^Key Laboratory of Instrument Science and Dynamic Test, North University of China, Taiyuan 030051, China

## Abstract

Context-aware user interface plays an important role in many human-computer Interaction tasks of location based services. Although spatial models for context-aware systems have been studied extensively, how to locate specific spatial information for users is still not well resolved, which is important in the mobile environment where location based services users are impeded by device limitations. Better context-aware human-computer interaction models of mobile location based services are needed not just to predict performance outcomes, such as whether people will be able to find the information needed to complete a human-computer interaction task, but to understand human processes that interact in spatial query, which will in turn inform the detailed design of better user interfaces in mobile location based services. In this study, a context-aware adaptive model for mobile location based services interface is proposed, which contains three major sections: purpose, adjustment, and adaptation. Based on this model we try to describe the process of user operation and interface adaptation clearly through the dynamic interaction between users and the interface. Then we show how the model applies users' demands in a complicated environment and suggested the feasibility by the experimental results.

## 1. Introduction

USA Scholar Schlit first proposed three target location based services (LBS) in 1994: spatial information, social information, and resources nearby. Nowadays, mobile LBS interface has significant influence, while a mass of users carry their mobile terminals to require location services such as the wish to find a better route all over. Under this circumstance, problems of traditional mobile LBS interface are revealed which lack of ability to adapt users' demands initiative. Context-aware human-computer interface makes mobile LBS interaction more natural and efficient which is able to adapt to different users' characters and requirements by taking advantage of usable information about users' tasks (e.g., locations and preferences of users, experiences, and surrounding environments). Many of these adaptive interfaces serve specific users, such as the interface which Cao designed for children that apply cartoon-icon in 2007 [1] and the user interface adaptation proposed by Zouhaier et al. which is based on context awareness for disabled people in 2013 [2], for the characters of specific users are obvious. And these researches are based on the human-computer interaction model which describes the characteristics of the interaction process between human and machine. Some early researches of adaptive interface model are based on user features, like Rich proposed a modeling method which classifies the users based on their background and then provide different services [3]. User modeling concentrated not only on users' cognitive or reason, such as knowledge, goals, and planning [4], but also on emotional and personality [5, 6]. In the field of mobile LBS, Shi and Bian developed an adaptive expression of spatial information and the adaptation policy of the interaction elements on LBS interface in 2007 [7] and Lathia et al. proposed state-of-the-art advanced traveler information system (ATIS) which can adapt to users' environment and activities in 2012 [8]. However, there are certain problems and shortcomings in the current study.

On the one hand, most of context-aware adaptive interfaces are designed for specific tasks or applications, and many researchers construct different models to satisfy users' various requirements that are ignoring the diversity of one person. Classification for users in one respect cannot represent the features on their other aspects. On the other hand, context-aware information is always used to predict the usable interface but ignores its effect in the dynamic interaction process. We maybe consider other aspects such as preference when we recommend a suitable interface to a user built on his/her cognitive ability. The challenge of adaptive interface in mobile LBS is not simply to provide users information whenever and wherever but also to provide appropriate information for users when they need. The current research on the interface adaptation lacks the exploration of user dynamic interactive behavior. When the passage of time, task, and context information change, then the content of adaptation changes. According to these problems, this paper proposed dynamic adaptive model and presents a corresponding method.

In view of the above problems, this paper presents a context-aware adaptive interface for mobile LBS. At first, we establish a user model which has better generalization and differentiation degree based on users' basic characteristic and the behavior characteristic, and then we match the user model with the refine interface element modules; proposed adaptive interface modeling method and system structure combine with dynamic interaction behavior. At last, we explain the adaptive process through a scenario. In the following Related Works section the feasibility and applicability of context-aware interface to be adapted to users in some solutions are discussed.

## 2. Related Works

This section shows the focus that we described below within context-aware adaptive interface for mobile LBS existing literature. There are three parts that we discussed: context-aware technology, adaptive user interface, and adaptive spatial information.

### 2.1. Context-Aware Information

People often naturally used implicit information to make the content rich when there is a process of human to human interaction for they understand the situation of each other while for computers it seems difficult to master this skill in comparison. Therefore, context-aware technology was used widely in order to attain the purpose of natural interaction. Context is determined by Merriam-Webster's collegiate dictionary as “the interrelated conditions in which something exists or occurs.” To put it more specifically, Schilit and Theimer [9] proposed that context contains location and identities of nearby people and objects in 1994, and in 1997 Brown et al. [10] added time of the day, season of the year, and temperature to the original definition. Up to now, context is broadening to a comprehensive concept including task context, user context, and circumstance context. Generally speaking, context based on mobile phones can be divided into three parts as follows [11]:

1. user environment: location, preference, experiment, social relations, and so forth;
2. mobile environment: device suitable for users to input or display, network, Bluetooth, and so forth;
3. physical environment: weather, date, noisy, and so forth.

Moreover, context-aware technology has the capability to sense, detect, and grab the environment around users and get the dynamic changes to speculate their behavior [12].

Context-aware technology plays an important role in mobile terminals which equip a rich set of sensors (e.g., camera, accelerometers, GPS, digital compass, gyroscope, ambient light sensors, proximity sensors, multitouch panels, and microphone) [13]; it also enriches the function of GIS to provide users a variety of services. Tomitsch et al. discussed the context of human actions in public space and how they fed back [14]. Lathia et al. [8] proposed mobile traveler information system which can become personalized services based on explicit preferences. J. Karat and C.-M. Karat [15] proposed context-aware route recognition approach to improve the accuracy of routing recognition. Abowd et al. proposed a mobile context-aware tour guide in 1997 [16]; Cai specifies a semantic model which combined with context and demonstrates how this model supports contextualized interpretation of vague spatial concepts during human-GIS interactions in 2007 [17]. Chung and Schmandt proposed a mobile user-aware route planner which can learn a user's everyday routes and provides directions from locations along those routes in 2008 [18].

### 2.2. Mobile Adaptive Interface

Human-computer interface (HCI), which is also known as the user interface, is media for the exchange of information between user and computer. The traditional design methods consider the efficiency problem of using rarely, and the traditional interface can only adapt to a few people, but also cannot meet the requirements for one person in different periods with the fixed user interface designed according to users' average level while the computer used popular and user group became more and more widely used. Adaptive user interface (AUI) which can adjust itself to fit a user or a task [19] emerged and developed fast while the requirement of omnipresent computing challenge traditional interface emerged and increased. Earlier in the research of an adaptive user interface, it requires three models: system model, user model, and the interaction model [20]. The system model describes the characteristics of the system that can be changed, such as the system to be able to adaptive. Acquisition and application of the user model are the foundation of an adaptive user interface to make the system adapt to the individual user behavior. Interaction model defines how the system is modified, and what it can adapt to. Above all, the degree of adaptability in the adaptive process depends on the user model which describes users' knowledge that can be utilized to facilitate human-computer interaction.

Many researches take advantage of adaptation to provide personalized services for users such as helping users to obtain information, giving users a recommendation, tailoring information for users, or providing help. Yoon et al. proposed an adaptive mixture-of-experts model to solve the complexity and personality problem of multiuser interface in 2012 [21]. And Cheng and Liu developed an adaptive recommendation system that inferred users' preferences and adjusted the user interfaces [22]. Wang et al. presented an automatic approach which helps users who suffer from visual impairment to make use of online map with independent access to geographic direction [23]. Sulaiman and Sohaimi [24] discuss a possible interface which is simple enough for older users through analyzing the situation of using a mobile phone.

### 2.3. Adaptive Spatial Information

Nowadays, people can carry mobile devices everywhere and every time with the development and extensive application of mobile communication and internet technology. In this case, a large amount of requirements concentrated on the interests of users themselves: environment information such as recommending interest point to users by acquiring the users' location. We often need to consider the personal issues for spatial information used by more and more users. In particular, the users which have mobile phones with different running speed needed different degrees of information presentation, and the users with unique moving speed needed different scale. In addition, different users request different aspects of spatial information; for example, tourists pay attention to scenic spots while drivers follow with road conditions. Another adaptive problem is how to determine quantity of information displayed on the screen. Many navigation charts are based on accessibility to display all the details when facing the problem. In fact, showing the map too detailed not only is difficult to understand and display but also makes the user focus on useless information which limits the effectiveness. Therefore, mobile adaptive visualization of spatial information must be based on users' needs to provide details step by step [25] such as providing user detailed information when he/she amplifies the map gradually.

In summary, the key elements of adaptive spatial information are related with the user, the mobile terminal, and the environment. User aspects include user background (such as physiological differences, preference differences, and cognitive differences), user location (position, speed, and direction), and user requirements. Environment aspects include basic information (such as temperature, weather, date, and time), and related users' information means the information of the other users which related to the users' tasks. And the effects of system contain transmission speed, information receiving rate, and so on.

Adaptive interface has been extensively studied, but the self-learning user interaction lacks. Here we adopt the use of adaptive user interface model to predict user's intent effectively with their spatial experience. Moreover, the proposed use of experience awareness assists in the prediction of user's intent while satisfying the early fire detection requirement.

## 3. Adaptive User Interface Model

Cognitive psychology regards people as an information processing system, and people often have different action according to the environment, cognition, and personality tendency differences to reach the established goals [26]. There are interaction spaces between human, computer, and environment. If the information presented on the interface can adapt to the user's cognitive psychology and personality traits, users will complete the task quickly for reducing the user operations.

Adaptive user interface model consists of the following components: user model (UM), task model (TM), interaction model (IM), domain model (DM), environment model (EM), and presentation model (PM).

### 3.1. User Model

The user model needs to abstract the individual differences of users which may relate with personalized service for no two users are identical. The interface style may be affected by personality or preference differences while the expression mode of interface may be affected by cognitive or physiological differences. Here we define the user model (UM) as a collection: UM = {*User ID, Knowledge, Physiology, Inclination*}.* User ID* represents a unique identifier of user. And we divide users' background into three parts:* Knowledge*,* Physiology*, and* Inclination*.* Knowledge* summarizes the knowledge level of the user,* Physiology* is on behalf of users' physiological characteristics, and* Inclination* represents the subjective desire in every aspects.

At first, people always associate academic quantification when it comes to knowledge, but we use the concept to represent users' skill level including proficiency in interface using and cognitive ability on the map. Knowledge = {*Education, Professional level, Proficiency level*}. And we can see the differences existed between expert users in a field on software proficiency through the description. Second, factors of physical abilities cannot be ignored in the interaction. Physiology = {*Age, Sex, Health*}. And last, Inclination = {*Occupation, Personality, Habit, Preference*}; these factors can influence the choice tendency of users.

### 3.2. Interface Static Elements

The task model was divided into abstract task model and specific task model. Catch and abstract the users' needs and described the needs as abstract tasks. Describe the interactive behavior in the system and the dynamic behavior in the process of the interaction. The task is an activity which is used in order to satisfy the user's goals. We can abstract task as TM = {*Operation, Object, TC*}. Operation means a task operation which act on an object. The object means an object which needs to operate. TC represents the task context. The task model can be embodied into STM = {*tID, operationType, dataItem, dataType, C*}.* tID* means the task ID which needs to complete. operationType is the type of interaction operation such as read, write, or command. dataItem is a data item which consists of data ID, data attributes, and data value. dataType means the data type of the operate data. *C* is a set of constraints to the data item. Specify the task context to the constraint of the data and data manipulation.

The domain model (DM) is defined as DM = {*Object, Attribute, Contact, Time*}; Object is a set of interface object at a specific time. The interface object is changing when the environment changes or user task changes; therefore we use Time which is corresponding with the Time of TM and EC to distinguish.

The interface model (IM) describes the interface, and express the various controls manipulation in the dynamic interactive process. Adjust the user interface components and structure according to the specific task which is analyzed through the task model. The interface model can be abstracted as IM = {*Control, controlConstraint, controlRelation*}. Control contains control ID and control attribute.* controlConstraint* means the constraints of the controls on the interface.* controlRelation* means the relationship of the controls on the interface.

The presentation model (PM) is defined as PM = {*Module, MEC, MUC, MTC*}; Module is a collection of interface components. MEC, MUC, and MTC are component properties under the influence of the environmental context, user context, and task context.

### 3.3. Interactive Context

We can divide interactive context (IC) into three parts: environment context (EC), user context (UC), and task context (TC). Interactive environment context can be divided into user environment and equipment environment, where the user environment includes time, place, and weather and equipment environment includes transmission rate, and resolution. EC includes the user environment and the device environment. The user environment includes perimeter environment which may affect operations of the user and user's own context environment. Device environment includes transmission rate and resolution. EC = {*DE, UE*}; DE means the device environment and UE means the user environment. DE = {*MS, SO, TR*}. MS means the movement speed of the device; SO is the screen orientation, and TR is transmission rate of the device. UE = {*Loc, Sur, Soc, Act*}. Loc means the location information of the user. Sur represents the surrounding users of the user. Soc means the social information of the user which can be obtained from the social software open interface. Act is the information which is provided by the previous operation.

### 3.4. Interaction Methods

A large amount of contextual information and the changeable situation requires a decision mechanism to determine what kind of context information is needed and when the information can be used. The decision mechanism has three points which must be paid attention to: selecting the appropriate context, allocating context priority level reasonable, and having the dynamic adaptability (environment-stimulation, task-stimulation, etc.).

The most important in the interaction process is to understand the purpose, and then we must reduce the problems caused in the process of achieving this goal; the last is to let the user interface elements fit the user like Figure 2.

The interaction model adjusts interface mode constantly when some factors dynamic changes and stimulates.

## 4. Interaction Strategy

The adaptive user interface framework which based on the GIS interface model is presented in the Figure 3. The Figure 3 describes the process of context information collection and the eventual capture, and explains how to analysis and handle events through the certain reasoning mechanism. According to psychology related research achievements, people will divide continuous events into several activities in perceptual according to kinds of characteristics. Individual differences in cognitive flexibility may underlie a variety of different user behaviors [27]. And users in the same activities tend to repeat steps which can reduce the user experience.

Combine the knowledge base to identify the user's interaction patterns and predict the most likely interaction behavior candidates of the user; then the adaptive recommendation results appeared on the interface layer. The adaptive user interface framework interconnected between layer and layer; the model layer determines the need of context information collection, the adaptive layer is used to realize the user interface adaptive function and scheduling, and the interface layer is used to present the results of self-adaptation.

The adaptive decision-making mechanism is implemented by capturing the user interaction sequence. When the same user completes a task on the mobile GIS interface, the same action sequence is often repeated. So we can predict the future behavior of the user through the interaction sequence judgment, storage, and matching. The tasks which were completed by the GIS human-computer interface can be refined, and the user operation of every subtask will have certain regularity which also contains its unique personalized information. User actions can be refined into lower levels of atomic operations, such as a button click, an operation of input box, and the map zoom event. We can describe a user action as A = (action object, action type). Several user actions compose an action sequence, which can be collected and matched to predict the most likely next step of users. Then adjust the interface elements dynamically and achieve the goal of continuously reducing the user operation.

In the process of matching the sequence, we judge the next action according to the front action, but the longer the length of the sequence matching, often the better the results. Therefore, we should choose more suitable length sequence to match. Defining the average length probability of the match sequence is *L*(*a*∣*s*) = *l*_*t*_(*a*, *s*)/∑_*i*_ *l*_*t*_(*a*_*i*_, *s*). In addition, the happening of the action may also be related to the other action and not just related to the matching of model length, so we use frequency of action occurrence *P*(*a*∣*s*) = *f*(*a*, *s*)/∑_*i*_ *f*(*a*_*i*_, *s*) to describe the action occurrence probability. We use the action prediction *R*_*t*_(*a*, *s*) = *L*(*a*∣*s*) + *P*(*a*∣*s*) to determine the action occurring possibility.

The interactive action sequences appearing occasionally can be seen as preinteraction pattern, which occurred repeatedly will be put into the pattern library. The current interaction sequence is matching with the action sequences library, and the matching starts from the current action and increases in length gradually under the context environment. Obtain all the forecast candidate set; then choose the action which has highest action evaluation *R*_max⁡_ as the prediction results like Figure 4. *E* means the new action set and *C* means the existing action set.

## 5. The Modeling Method

In the third part of the paper the adaptive user interface model is put forward and this section will illustrate the construction process from the abstract model to the specific model. Create an adaptive interface model on the basis of the user model, extract the information from the user model to form the domain model, and then extract the management tasks in the field of domain model to form the task model.

**Figure 1:**
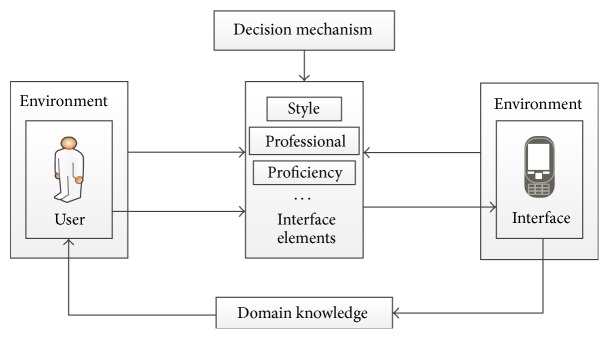
The environment of interaction.

**Figure 2:**
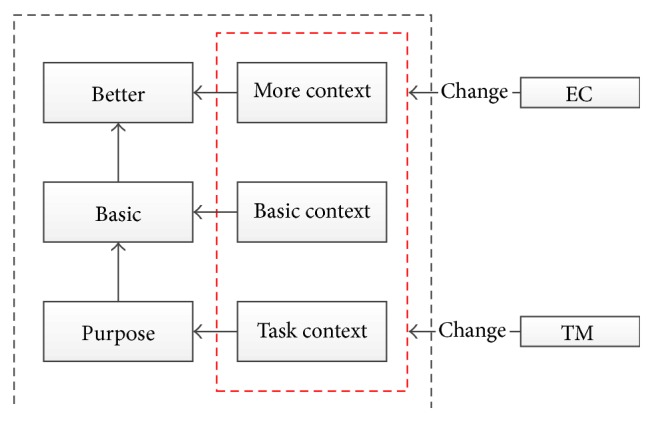
User interface adaptive elements.

**Figure 3:**
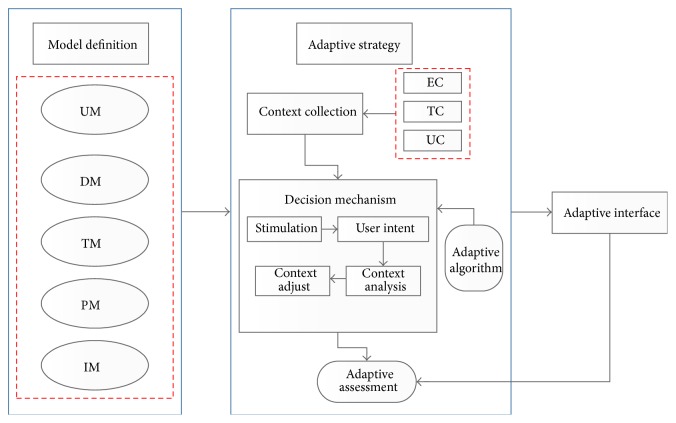
Mainly adaptive system.

**Figure 4:**
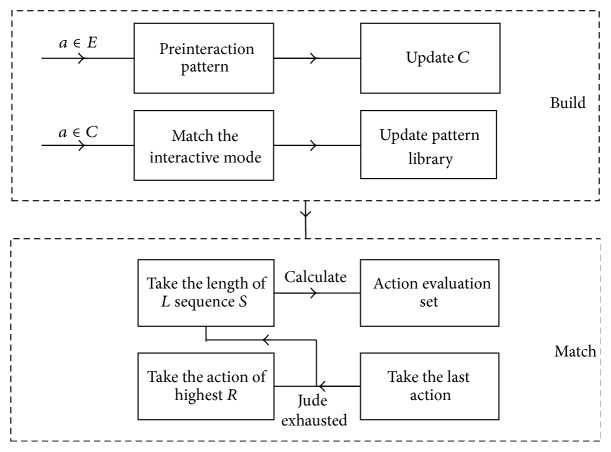
Sequence matching.

The first step is to build a user model. The user ID here refers to the account of each user in the system which is used to record different user information. Knowledge here means the proficiency of the user used navigation software and professional level of the user (Figure 1). Different levels of users can lead to different operations. And the Physiology refers to the aspects of users' age and gender differences. We will comprehensive considering these aspects in the experimental personnel selection. Inclination information is recorded automatically in the system.

Domain model which is considered from the user model needs to list managerial entity objects and analyze the properties of these entity objects. For example, when a user finds the route, entities in the domain include buttons, input box, and map, and the interface elements of other services may be involved for different users. The relationship between the interface elements is the ordinal relation in the operating sequence which is described in the adaptive strategies.

Each interact action corresponds to a task in the task model, such as the switch interface, input box operation, and determining button click. For example, a user wanted to find the point of restaurant; the description of the scenario in the user model can be defined as US = 〈(UserID1, Preference), (Device1, UserEn1)〉, UserEn1 = 〈Location, restaurants, Pre-operation〉; the corresponding task model can be defined as Task = 〈AT1, AT2, AT3〉, AT1 = 〈Click, ButtonStart, TC1〉, AT2 = 〈Click, ButtonSelect, TC2〉, AT3 = 〈Zoom, Map, TC3〉, TC = 〈Pre-operation, OPtime, Null〉.

The building of interaction model according to each task of the set of events in the task model described the atomic operations in the user interface such as clicking on and long press and described the corresponding commands of interacting objects, such as a jump and zooming.

## 6. Experiment and Analysis

### 6.1. The Scene

In order to verify the result of the study, the following scenario is designed to verify that the adaptive method is easy to use. And then we evaluate some values which are measurable.

*A Traditional Route and POI Searching Is as Follow*

1. User enters the application.
2. Go to the weather page to check the weather.
3. Go back to the main page.
4. Click the button to enter the route searching page.
5. Enter the locations to which the user wants to go.
6. Click the button to choose transportation.
7. Click the button to display the route.
8. Complete the route lookup.
9. Click the button to enter the POI selection page.
10. Choose a specific point of interest.
11. The points of interest appeared on the map.
12. Choose one of them to show the route.
13. Complete the POI searching.

*A Traditional Map Operation Is as Follows*

1. User logs into the application.
2. User clicks the search button.
3. Enter the search site in the input box.
4. Click the confirm button.
5. Zoom in to check the location
6. Zoom in once again.
7. Click on the map sign to check the specific location name.

*Context-Aware Adaptive Interface for Route and POI Searching Is as Follows*

1. User clicks the traffic mode button (preferences record) to enter the application.
2. Choose the traffic mode (advice according to the weather condition) and enter the application.
3. Enter the locations to which the user wants to go.
4. Click the button to display the route.
5. Complete the route lookup.
6. Click the button (preferences record) to show the points of interest.
7. Choose one of them to show the route.
8. Complete the POI searching.

*Context-Aware Adaptive Interface for Map Operation Is as Follows*

1. User logs into the application.
2. User clicks the search button.
3. Enter the search site in the input box.
4. Zoom in to check the location
5. Click on the map sign to check the specific location name.

Analysis of these scenarios can be found that, the traditional routes and POI searching need more options, for more user active choices, which will produce more returns and select operation. Here is the design of context-aware adaptive interface which is user centered (Table 1).

**Figure 5:**
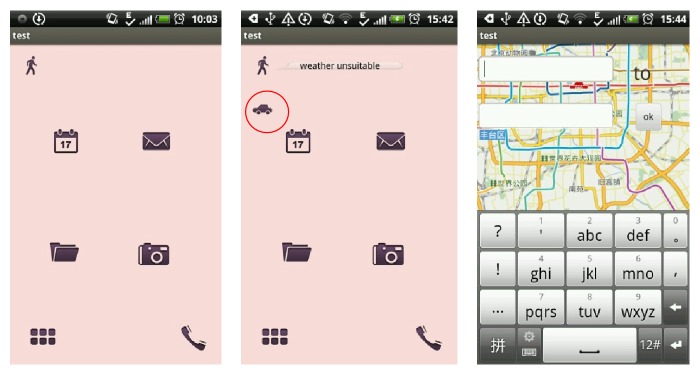
Route searching.

Figure 5 shows the route searching interface. The application icon is displayed as traffic mode according to user's preferences. It will prompt the weather condition when user clicks the button and give advice. Click on the icon to enter the location input interface. The context-aware adaption can reduce the traffic mode selection operation and also give advice according to the environment actively.

**Figure 6:**
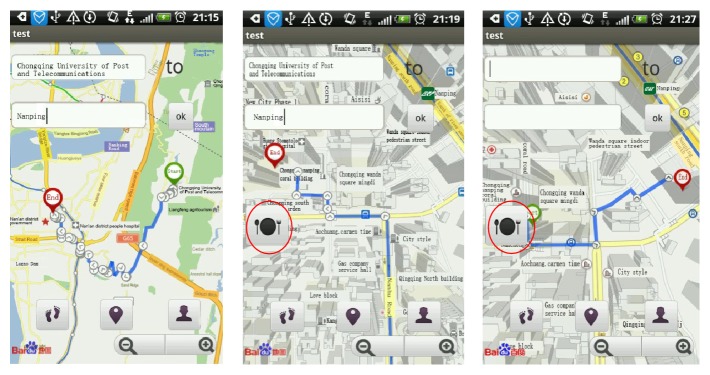
POI searching.

Figure 6 shows the adaption of interesting point searching. In the process of user walking, finding interesting point and giving corresponding button to users according to the preferences can meet users' requirements more easily. Click the button to access the route.

**Figure 7:**
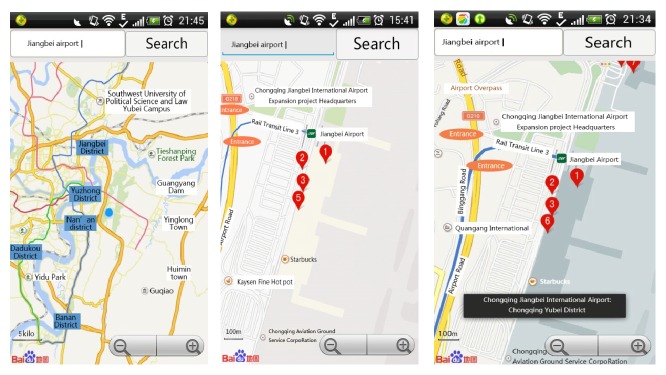
Map operation.

Figure 7 shows that the system recorded the user action sequence and forecasted the next steps. The operation sequence frequently appearing of the user in this scenario is 〈1, liu, searchBtn, click, time1〉, 〈2, liu, Inputbox, input, time2〉, 〈3, liu, OkBtn, click, time3〉, 〈4, liu, Map, zoom  in, time4〉, 〈5, liu, Map, zoom  in, time5〉, 〈6, liu, Map, click, time5〉. The system matched the first three operations and then predicted the next operation and adjusted the interface automatically. The first figure shows entering the search interface after clicking the search button, the second figure shows the amplifying map automatically after clicking the Ok button, and the third figure shows the location information after clicking the site.

We reflect the dynamic adaptive from two aspects mainly from this experiment: the choice of transportation mode and the user's interest concerns.

### 6.2. User Evaluation and Analysis

We choose some test users with certain discrimination and finish the appointed tasks. We choose 50 testers by taking the differences of users into consideration in the user model. We choose half of the testers' education level such that it is above the average and the other half is below the average. Including the testers, the proficiency can be divided into skilled, general, and strange. The sex ratio is 1.5 : 1 and age distribution from 20 to 60 years old, which were randomly selected.

The international organization for standardization (ISO) includes the usability evaluation factors of a product which fixed tasks in a specific environment which are effectiveness, interaction efficiency, and user satisfaction. The effectiveness is used to judge whether it can achieve certain functions and interface supports the corresponding function. There are two functions of the testing interface: providing the transportation recommended automatically and providing the interest recommendation when the user finds route. The user satisfaction is the subjective satisfaction of the user interface. The evaluations of these two aspects are assessed by the user survey. Interaction efficiency is decided by error rate, completion time, being easy to learn, and being easy to use. We can record the error time and completion time of two tasks and obtain testers' evaluations about being memorable, easy to learn, and easy to use and the efficiency. We let the testers to complete two contrast tasks under the same condition and get some pairs of observe values. Analyze these values to draw inferences. The difference result which is gotten from same testers in the same environment can be regarded as the differences made by different system. We use general navigation system to do the comparison test and obtain independent observations in pairs.

*X*_*i*_ represents the time spent by comparison system to complete a task, and *Y*_*i*_ represents the time spent by test system to complete a task. Suppose there are *n* pairs of independent observations: (*X*_*i*_, *Y*_*i*_)  (1 ≤ *i* ≤ *n*). *D*_*i*_ = *X*_*i*_ − *Y*_*i*_  (1 ≤ *i* ≤ *n*) is the difference of *X*_*i*_ and *Y*_*i*_; observations of these values' sample mean and sample variances are recorded as $\bar{d}$ and *s*_*d*_^2^. The rejection region is $\left|t\right|=\left|\bar{d}/\left(s_{d}/\sqrt{n}\right)\right|\geq t_{a/2}\left(n-1\right)$.

The first task is to choose a vehicle and find the route to reach somewhere. The second task is finding interest point around. And the third task is the map operation. At the beginning of the experiment stage let the users be familiar with the system and task. Record the time of each task completed and the time of total task completed in the course of the experiment. Count the total number of errors which emerged in the processes of the tests using. And let each test personnel complete the questionnaire at the end of the experiment. The time of users, which are familiar with this kind software, to complete the first task is usually about 23 s, and the time of users to find the corresponding route by using the system which records the users' selection and offers suggestions is about 16 s. These times are average values of testers. For the first task $\bar{d}\approx 6$, *s*_*d*_ ≈ 40.714, *t*_0.05_(49) = 1.6794, |*t*| ≈ 1.032 which are out of the rejection region. For the second task $\bar{d}\approx 7$, *s*_*d*_ ≈ 26.589, *t*_0.05_(49) = 1.6794, |*t*| = 1.8429 which fall into the rejection region and $\bar{d}\approx 5$, *s*_*d*_ ≈ 20.533, *t*_0.05_(49) = 1.6794, |*t*| = 1.7046 which fall into the rejection. The fundamental task needs less average time, but the advantage is not obvious through the calculation. In the second task and the third task, operating time reduces significantly through the calculation. The error rate has little difference between the contrast system and the test system. We can see that other factors are higher than contrast system except memorability. In addition, efficiency of the test system improves obviously.

**Figure 8:**
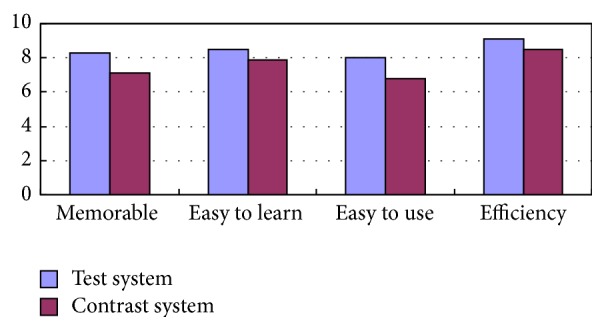
Questionnaire statistics.

Then we get the user's satisfaction degree of the interface on the aspects like being memorable, easy to learn, and easy to use and efficiency through the questionnaire survey. These 50 testers give the scores of two systems using experience and get the average of each index, respectively, like Figure 8. We can see that memorability of the traditional human-computer interface is higher than the adaptive interface for elements of adaptive human-computer interface are changeable. On the other aspect, the scores of text system are higher than the contrast system which efficiency is greatly improved. To illustrate the adaptation of the text system is greatly improved.

## 7. Conclusion

This paper proposes a context-aware adaptive human-computer interface model for mobile LBS which is based on the user model and described in three aspects: static composed elements, dynamic interactive behavior, and adaptive strategy. The adaptive user interface proposed in this paper has advantages compared with traditional adaptive user interface as follows: (1) avoiding the limitation of the traditional adaptive user interface caused by user classification and achieving the adaptation according to the combination of each user's habits and external experiments; (2) paying more attention to the dynamic interaction process and adjusting the user interface in the interactive process more in line with the real-time interaction; (3) using the context information dynamically and then making the context information using more effective.

The adaptive system based on the model in this paper has some deficiency; for example, the range of adaption should be extended, and also there are limitations of the current research to gain more effective information on user knowledge, ability, and so on. In the future, we will further optimize the stimulus-judgment method, more effectively use context information, enhance the adaptive result, and improve the interface layout mechanism to reach the goal of smooth and natural interface adaptive.

## Figures and Tables

**Table 1:** Context-aware adaptive interface scene design.

User	System
(1) User clicks the traffic mode button.	(1) Display “bad weather”; recommend another traffic mode.
(2) User chooses traffic mode and clicks the button.	(2) Display the location input box.
(3) User inputs the location and clicks the OK button.	(3) Show the route and the interest point button.
(4) User clicks the interest point button.	(4) Display the points of interest.
(5) User clicks the interest point.	(5) Display the route.
(6) User logs into the application.	(6) Record the user's operation.
(7) User clicks the search button.	(7) Enter the search interface.
(8) Enter the search site in the input box.	(8) Operation sequence matching and display location markers directly.
(9) Zoom in to check the location.	(9) Operation sequence matching and zoom in again.
(10) Click the map sign.	(10) Display the specific location name.
